# Cytoskeletal and phagolysosomal failure in macrophage antibacterial immunity: the PRRSV–porcine alveolar macrophage axis as a natural-host model

**DOI:** 10.3389/fimmu.2026.1892313

**Published:** 2026-10-14

**Authors:** Yubo Wang, Yihao Shang, Huiyang Yu, Xintong Li, Ailin Ji, Liang Wang, Jilin Chen, Yu Qi

**Affiliations:** 1 Department of Clinical Laboratory, The First Affiliated Hospital of Dalian Medical University, Dalian, China; 2 Department of Comparative Medicine, Dalian Medical University, Dalian, China; 3 Department of Nephrology, The First Affiliated Hospital of Dalian Medical University, Dalian, China

**Keywords:** host–pathogen interaction, innate immunity, macrophage antibacterial immunity, phagolysosomal killing, porcine alveolar macrophages, professional phagocytes, PRRSV, viral–bacterial coinfection

## Abstract

Viral infection of professional phagocytes can compromise antibacterial immunity because the cellular machinery reshaped for viral replication and immune evasion overlaps with the machinery required for bacterial capture, intracellular killing, and inflammatory signaling. Phagocyte cell biology has defined core cytoskeletal and trafficking nodes, including Rho-family GTPase control of actin assembly, integrin-mediated adhesion, and Rab-dependent phagosome maturation, and shown how bacterial pathogens target these nodes to evade clearance. Here we ask how recently described porcine reproductive and respiratory syndrome virus (PRRSV) lesions in porcine alveolar macrophages (PAMs) fit into this established cell biology. Direct evidence supports reduced phagocytic capture and impaired intracellular bacterial killing in defined systems. *In vivo* coinfection studies also report worse outcomes. Enhanced association or internalization of selected bacteria has also been reported, with ITGα5 and filamin A upregulation proposed as a candidate explanation. PRRSV further disrupts innate-immune signaling, autophagy–lysosome trafficking, ER–mitochondrial metabolism, and lipid homeostasis. These changes may contribute to antibacterial failure, but bacterial endpoints were not measured in the same experiments. At the tissue level, macrophage attrition and altered bystander states may disrupt cytokine relay, creating a permissive lung environment even when only a minority of PAMs are productively infected. PRRSV is particularly informative because it productively infects a professional phagocyte in the natural host, allowing viral perturbation, macrophage antibacterial function, and bacterial challenge to be examined in one tractable system. Because the candidate nsp5–RhoA trafficking route and the ITGα5/FLNA entry route were reported in different experimental systems, their potential combination defines a testable hypothesis requiring co-validation in the same primary PAM system. We use the PRRSV–PAM axis as a natural-host model to examine how viral infection of professional phagocytes can produce macrophage antibacterial failure, with implications for virus-associated bacterial vulnerability in veterinary and human respiratory disease.

## 1 Introduction

Viral infection of professional phagocytes creates a structural conflict: the machinery reshaped for viral replication and immune evasion overlaps with the machinery used to capture and kill bacteria and to coordinate inflammatory relay. This conflict can reflect direct competition for shared resources or collateral damage when viral perturbations disable host nodes that also support antibacterial defense. Current PRRSV evidence is more consistent with collateral damage operating at both tissue and cell-intrinsic scales. The nsp5–RhoA pathway is one candidate example: PRRSV-dependent RhoA/F-actin loss and RhoA-dependent antibacterial trafficking were demonstrated mainly in an immortalized porcine macrophage system, with selected molecular steps confirmed in primary PAMs (1).

The PRRSV–PAM axis provides a natural-host model in which productive viral infection, macrophage antibacterial function, and bacterial coinfection can be examined together. PRRSV replicates productively in the porcine alveolar macrophage (PAM) (2), with entry mediated by the scavenger receptor CD163 and ancillary receptors (3–5). Productive infection enables direct exposure to viral effectors such as nsp5, whereas receptor-mediated capture impairment can occur even without productive infection (6). Genetic tools [CD163-edited pigs (7–11)], defined coinfection models, and tractable *in vivo* readouts make PRRSV–PAM an informative system for this question.

PRRSV ranks among the most economically damaging pathogens in global swine production, yet much of its burden comes from secondary bacterial disease (2). PRRSV-positive herds experience more frequent and severe infections with *Glaesserella parasuis*, *Streptococcus suis*, and *Actinobacillus pleuropneumoniae* (12–16), though the predisposition is not uniform: NEB-1 did not consistently potentiate bacteria in SPF pigs (17), whereas controlled coinfection studies with highly pathogenic strains document worse outcomes across multiple pathogen–strain combinations (18). The outcome depends on the assay, because particle uptake, intracellular killing, and whole-animal coinfection interrogate different defense steps (6, 19–21). The pattern parallels post-viral bacterial superinfection in human respiratory medicine, where alveolar macrophage reprogramming by influenza or SARS-CoV-2 similarly predisposes to bacterial pneumonia (22–29).

Existing PRRSV reviews survey immune evasion, inflammatory injury, autophagy, macrophage polarization, and vaccine biology (2, 30–35). The present Review instead organizes PRRSV-associated lesions along the functional sequence of macrophage antibacterial defense and asks which changes have been linked to defined antibacterial endpoints. It places the recent cytoskeletal findings in the context of Rho-family GTPase control of phagocytic-cup formation and phagosome maturation (38–40), bacterial subversion of the same host nodes (41, 42), and macrophage cytoskeletal regulation of antibacterial function (43). This endpoint-centered approach distinguishes shared phagocyte biology from PRRSV-specific lesions and complements broader reviews of virus-driven macrophage programming and respiratory-virus bacterial superinfection (36, 37).

Two limitations apply throughout. First, the mechanistic evidence is weighted toward PRRSV-2; the PRRSV-1 evidence discussed here is comparatively limited and includes a phagocytic study in primary PAMs (6). Second, although the PRRSV–PAM relationship provides a natural-host biological context, many individual mechanistic steps were established in immortalized or heterologous systems, including MARC-145 cells, 3D4/21 porcine macrophages, and HEK-293T overexpression systems. Their relevance to primary PAMs should not be assumed. Single-cell transcriptomic approaches can resolve macrophage heterogeneity but do not replace mechanistic validation in primary PAMs (44). Unless otherwise specified, PRRSV refers to PRRSV-2, which accounts for most of the mechanistic evidence reviewed here.

Literature searches were last updated on 27 August 2026. We prioritized mechanistic studies published from 2020 onward and included earlier foundational studies identified through PubMed, Google Scholar, and citation tracking. This is a narrative rather than a systematic review.

Throughout this Review, particle uptake, bacterial association, bacterial internalization, intracellular survival, directly measured killing, and whole-animal bacterial burden or pathology are treated as distinct, assay-specific outcomes. For each mechanistic claim, we identify the supporting experimental system, including pig *in vivo* studies, primary PAMs, immortalized porcine macrophages, MARC-145 cells, HEK-293T overexpression systems, and cross-species models. “Direct” refers only to the endpoint measured in the cited experiment. A route is described as a candidate mechanism when its causal chain is incomplete or has not been reproduced in primary PAMs; consequences extrapolated across cell types, pathogens, or experimental systems are labeled inferred or hypothetical.

## 2 Antibacterial failure at cellular and population levels

Macrophage antibacterial defense can be read as a sequence of functional checkpoints: capture of extracellular bacteria, maturation of bacteria-containing phagosomes, lysosomal killing, and inflammatory relay to recruit additional immune cells. The PRRSV literature is most useful when organized along this functional sequence rather than by viral protein alone. These checkpoints operate within individual cells and across the macrophage population; failure at either scale can weaken lung antibacterial defense.

Multiple independent laboratories, working with different PRRSV strains, bacterial species, cell systems, and assay formats, document a set of antibacterial defects (**Figure 1**). Across these studies, recurring defects include reduced phagocytic capture, impaired intracellular bacterial killing, and worse coinfection outcomes *in vivo*. Enhanced bacterial association has also been reported, although its molecular basis rests on fewer studies. Together, these defects form a working model in which PRRSV can compromise several distinct steps in the macrophage’s antibacterial sequence. This phenotypic foundation, converging across three decades and multiple independent laboratories on impaired capture (6, 19, 45), reduced oxidative burst (21), impaired killing of *G. parasuis* (46), and enhanced bacterial burden during coinfection (13–16, 47), is the empirical basis for the testable models discussed below.

**Figure 1:**
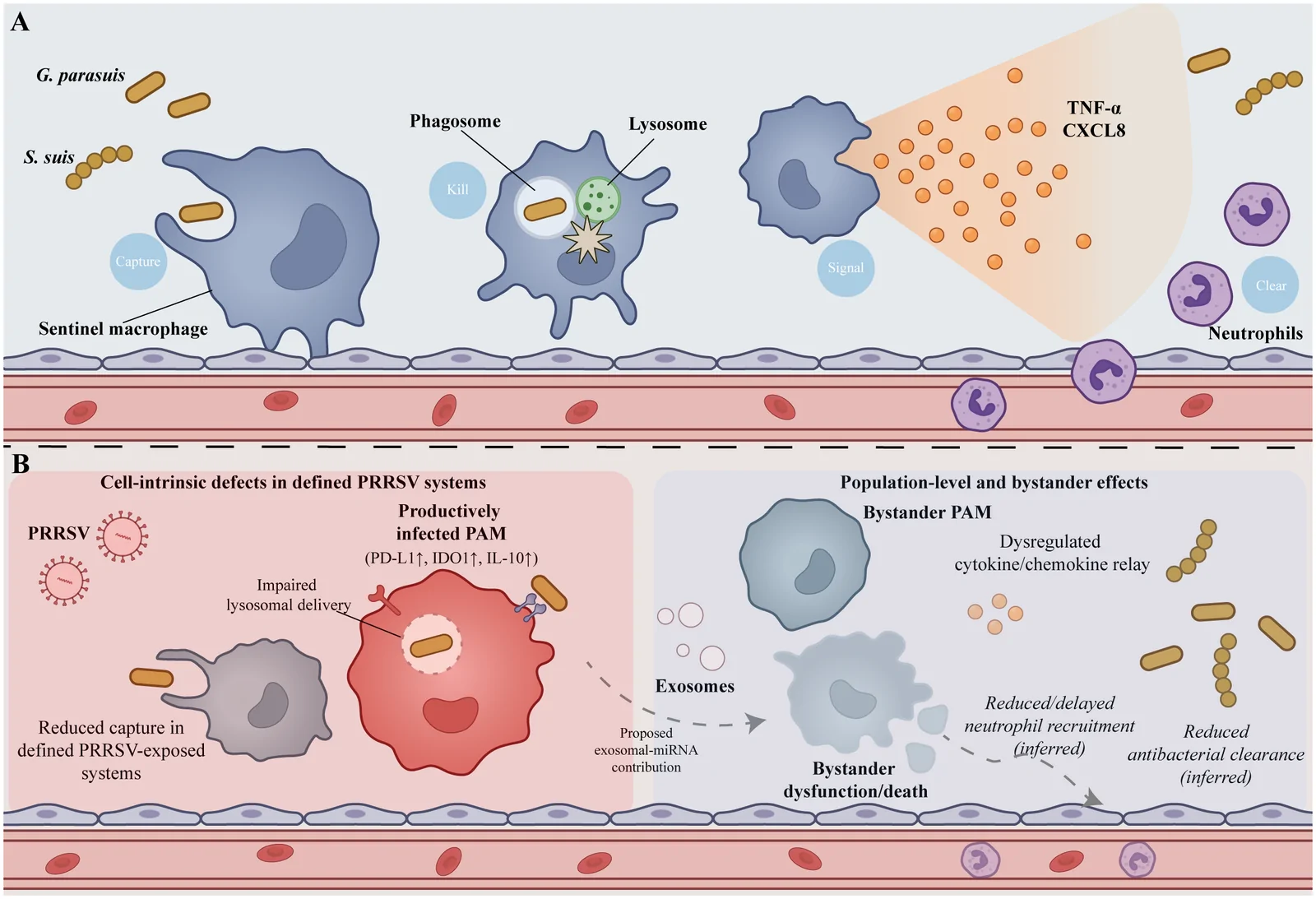
Antibacterial failure during PRRSV infection operates at cellular and population levels. **(A)** In the healthy airway, porcine alveolar macrophages (PAMs) capture inhaled bacteria, deliver bacteria-containing phagosomes to lysosomes for acidification and killing, and release cytokines and chemokines that recruit additional effector cells. These are distinct endpoints: capture does not establish intracellular killing, and either cellular endpoint can differ from whole-animal bacterial burden. **(B)** PRRSV exposure can weaken defense at two scales. At the cell-intrinsic level, reduced particle capture and impaired bacterial killing have been measured in PRRSV-exposed macrophages or pig pulmonary-clearance models (6, 19–21, 45, 46). nsp5–RhoA and ITGα5/FLNA are candidate explanations for lysosomal-delivery failure and selective bacterial entry, respectively (1, 57); their hypothesized integration is shown in **Figure 3**. At the population level, macrophage attrition, virulence-dependent population shifts, altered bystander states, and dysregulated cytokine relay may reduce the availability and coordination of antibacterial cells. In one PRRSV-2 BALF single-cell dataset comparing the JA142 and NA10 strains, viral tracking classified fewer than 5% of macrophages as directly infected at the sampled time points, with the highest counts at JA142–7 dpi and NA10–3 dpi (53); this criterion- and time-dependent proportion is not a general PAM infection rate. Productively infected PAMs preferentially upregulate PD-L1, IDO1, and IL-10 relative to bystander cells (54). Exosomal microRNAs have been proposed as mediators of bystander cell death on the basis of expression profiles and predicted targets, but causality has not been demonstrated (53). In panel B, the labeled cell-intrinsic defects within the pink compartment denote changes measured in the cited PRRSV systems. Gray dashed arrows denote inferred antibacterial consequences or proposed mediators lacking a paired causal test.

### 2.1 Impaired bacterial capture and uptake in viable macrophages

The first checkpoint is whether PRRSV-exposed macrophages can capture extracellular bacteria or bacterial-sized particles efficiently. Evidence that PRRSV impairs bacterial capture predates recent molecular studies. In a study primarily examining tulathromycin’s immunomodulatory effects, Desmonts de Lamache et al. also found that PRRSV infection reduced uptake of both non-opsonized zymosan and opsonized beads in porcine monocyte-derived macrophages (45). Thanawongnuwech et al. reported that a highly virulent PRRSV strain (VR-2385) reduced pulmonary particle clearance, though the effect was less pronounced with a modified-live vaccine strain (19). Neither study measured live bacterial survival, but both document impairment of pulmonary particle clearance capacity.

De Baere et al. extended these findings to primary PAMs infected with a European genotype (PRRSV-1) strain, showing that virion interaction with sialoadhesin is sufficient to inhibit bead phagocytosis even without productive infection (6).

### 2.2 Impaired phagolysosomal delivery and intracellular killing

Once bacteria are internalized, the next checkpoint is whether macrophages can deliver bacteria-containing phagosomes to lysosomes and execute intracellular killing. The available studies converge on a post-entry antibacterial defect, although they use complementary rather than equivalent endpoints. Solano et al. showed directly that *ex vivo* PAMs from PRRSV-infected piglets killed *Haemophilus parasuis* (*H. parasuis*; now reclassified as *Glaesserella parasuis*) less efficiently at 7 days post-infection (46). Thanawongnuwech et al. reported a 69.4% reduction in stimulated superoxide anion production in PAMs (P < 0.01) and a trend toward reduced bactericidal activity (21), supporting impaired effector capacity rather than providing a second definitive killing assay. Together, these findings support impaired post-entry antibacterial capacity and motivate the candidate molecular pathway discussed below.

Zheng et al. connected PRRSV-2 infection to intracellular bacterial survival through complementary experiments conducted mainly in immortalized PAMs, with selected molecular validation in primary PAMs (1). PRRSV-2 nsp5 recruited Smurf1 and promoted K63-linked ubiquitination and TOLLIP-dependent autophagy–lysosome degradation of RhoA. PRRSV-2 infection reduced F-actin and bacteria–lysosome colocalization and increased viable intracellular bacteria; *Aerococcus viridans* was the principal model, with confirmatory PRRSV-infection experiments for *Streptococcus suis* serotype 2 and *Klebsiella pneumoniae* (1). Separate *A. viridans* challenges established RhoA function: knockdown reduced F-actin and lysosomal targeting and increased viable bacteria, whereas overexpression produced reciprocal effects. The TOLLIP step is mechanistically congruent with its established role as a selective-autophagy adapter for ubiquitin-tagged cargo in other cellular contexts (48).

In normal macrophages, Rho-family GTPases coordinate actin remodeling during bacterial uptake and phagosome maturation (38, 39). In the PRRSV system, nsp5 diverts RhoA through a specific degradation route: Smurf1 attaches K63-linked ubiquitin chains that direct RhoA to the autophagy–lysosome pathway rather than the proteasome. RhoA depletion reduced F-actin and bacteria–lysosome colocalization and increased viable intracellular bacteria in immortalized PAMs, while the nsp5–Smurf1–RhoA interaction and endogenous RhoA degradation were also examined in primary PAMs (1).

Together, the paired loss- and gain-of-function data make the nsp5–RhoA axis the strongest current candidate molecular explanation for PRRSV-associated killing failure. The study connected PRRSV to RhoA/F-actin loss and separately established RhoA-dependent antibacterial trafficking in the same principal immortalized macrophage model. The defining next experiment is RhoA restoration during PRRSV–bacterial coinfection in primary PAMs across representative viral strains and clinically relevant bacteria.

### 2.3 Cell death, macrophage attrition, and cytokine-relay failure

Oleksiewicz and Nielsen reached a related but distinct conclusion: at 24 hours, acute infection did not reduce *E. coli* uptake by viable macrophages, but by 48 hours the defect reflected attrition of functional cells through PRRSV-induced cytotoxicity instead of uniform collapse of per-cell capacity (20). These studies reveal at least two routes to impaired capture: immediate receptor-linked suppression and later loss of functional macrophages. Thus, PRRSV can compromise antibacterial defense before intracellular killing begins, either by directly suppressing phagocytic uptake or by reducing the pool of functional macrophages available for capture. Immediate receptor-linked capture inhibition and later macrophage attrition are therefore distinct lesions; neither establishes uniform loss of phagocytic competence in every surviving PAM.

Cell-death susceptibility also varies with macrophage origin and experimental context. In the PRRSV–*Glaesserella parasuis* study by Kavanová et al., monocyte-derived macrophages showed substantially greater mortality and stronger induction of pro-apoptotic transcripts than PAMs, which were comparatively resistant under the same conditions (49). Concurrent bacterial exposure did not uniformly amplify these responses. PRRSV can also activate NLRP3-dependent pyroptosis in PAMs (50–52), but these studies measured inflammasome activation and host-cell death rather than bacterial clearance. They establish additional routes by which the functional macrophage pool may contract; they do not show that apoptosis, pyroptosis, and general cytotoxicity make equivalent contributions to secondary infection. Reported differences between productively infected and bystander cells further indicate population heterogeneity (53, 54), although a defined bystander pathway has not yet been shown to cause defective bacterial clearance.

PRRSV targets multiple pattern-recognition and inflammatory modules, but their relevance to antibacterial defense differs by endpoint. The most immediate link is altered cytokine output, a proximal component of the relay that recruits and activates additional effector cells. During concurrent PRRSV and *Glaesserella parasuis* infection of monocyte-derived macrophages, IL-1β and CXCL8/IL-8 expression was lower than after either infection alone, and experimental IFN-α exposure reproduced part of this suppression (55). These experiments establish altered cytokine output; effects on neutrophil recruitment and bacterial clearance remain to be tested directly.

The inflammatory phenotype is not simple silencing. A PRRSV-2 double mutant with altered nsp1β-mediated IFN antagonism and N-mediated NF-κB activation produced less inflammatory cytokine during *Streptococcus suis* coinfection and caused milder disease than the wild-type virus (56). The mutant also had lower viral titers and a shorter duration of viremia, so the experiment does not isolate cytokine output as the sole cause of the improved bacterial coinfection outcome. The experiment instead shows that the balance between antiviral signaling and inflammatory amplification contributes to disease severity. Cytokine suppression can delay antimicrobial recruitment, whereas excessive inflammation can damage the respiratory environment in which clearance must occur.

Together, cumulative attrition, subpopulation targeting, and sentinel-relay failure support a plausible, testable population-scale model linking low cross-sectional infection rates to severe antibacterial dysfunction. Resolving the contribution of this route relative to cell-intrinsic killing failure requires matched primary-PAM and *in vivo* experiments that measure viable macrophage abundance, cytokine protein output, bacterial capture, and intracellular killing in parallel.

### 2.4 Selective bacterial association and entry

A second, apparently opposite, phenotype is selective bacterial entry enhancement; this should be distinguished from bulk phagocytic capture measured with beads or particles. Multiple *in vivo* coinfection studies document increased bacterial burden in PRRSV-infected animals (13–16, 47), and an NADC30-like strain accelerated *S. suis* invasion both *in vivo* and *in vitro* (47). Together with the mechanistic entry study described below, these observations raise the possibility that increased bacterial association contributes to the phenotype, beyond what impaired killing alone would predict. A recent study offers a candidate molecular mechanism for this phenotype.

Liu et al. combined primary PAM, 3D4/21, MARC-145, piglet, and mouse experiments (57). In cell systems, PRRSV-2 increased ITGα5 expression and FLNA abundance, and perturbing this axis altered bacterial adhesion or invasion. Piglet experiments linked PRRSV challenge to increased bacterial burden during coinfection. Separately, lentiviral shRNA knockdown of FLNA or ITGα5 reduced bacterial lung infection and mortality in mice challenged with bacteria but not PRRSV; this supports host-factor relevance to bacterial infection rather than *in vivo* validation of a PRRSV-specific mechanism. Enhanced entry was demonstrated for *Streptococcus suis* serotype 2, *Klebsiella pneumoniae*, and *Aerococcus viridans*, whereas *Staphylococcus aureus* served as a negative control (57). The proposed HOXD8→ITGα5 transcriptional step remains a focused target for independent validation because it currently lacks non-PRRSV precedent.

The ITGα5–FLNA phenotype addresses a different endpoint from generalized capture impairment. PRRSV could increase association or internalization of selected bacteria even while bulk particle phagocytosis declines. ITGα5 is a fibronectin receptor, and FLNA organizes cortical actin that supports integrin clustering; together they provide a plausible host-side interface for bacterial docking (57). The proposed fibronectin bridge, the nsp10–HOXD8 regulatory step, and the relationship between this entry arm and RhoA activity have not been closed in the same primary PAM. Increased entry would favor bacteria only if the organisms also avoid or outlast downstream killing.

### 2.5 Pathogen-, strain-, time-, and endpoint-specific boundaries

The functional relevance of capture defects, entry enhancement, and killing failure depends on bacterial lifestyle, PRRSV strain virulence, infection timing, macrophage origin, and the endpoint measured. Defined animal models show increased susceptibility to *Streptococcus suis* or greater bacterial burden under particular challenge conditions (13–16, 47), while macrophage studies show that concurrent PRRSV and *Glaesserella parasuis* exposure can alter inflammatory outputs (55). Kavanová et al. did not establish a uniform PRRSV-induced apoptosis phenotype in PAMs: monocyte-derived macrophages showed greater mortality and stronger induction of pro-apoptotic transcripts, whereas PAMs were comparatively resistant; multinucleated giant cells were observed only in *G. parasuis*-exposed monocyte-derived macrophages (49). These findings cannot be pooled across macrophage origins or treated as direct evidence of failed bacterial clearance.

Discordant evidence also belongs in this analysis. Cooper et al. found that NEB-1 did not consistently potentiate secondary infection by *G. parasuis*, *S. suis*, *Salmonella cholerasuis*, or *Pasteurella multocida* in SPF pigs (17). The most defensible statement is therefore that PRRSV predisposes pigs to bacterial disease under defined strain–pathogen–endpoint combinations, not uniformly.

Bacterial lifestyle determines which failure mode is most relevant. Organisms capable of conditional intracellular persistence may benefit from a combination of selective entry and impaired killing, whereas predominantly extracellular pathogens can benefit from delayed capture, macrophage attrition, or failed cytokine relay without occupying a stable intracellular niche. These are not mutually exclusive models, and animal bacterial burden cannot identify which cellular route predominated. The two candidate molecular arms also remain independently supported rather than experimentally integrated (1, 57).

These dependencies are reflected in several specific findings. Cooper et al. (17) reported no potentiation with NEB-1 in SPF pigs, whereas Brockmeier et al. (18) documented worse outcomes with highly pathogenic Asian isolates, indicating that strain virulence is a primary determinant of coinfection outcome rather than PRRSV infection per se. Solano et al. reported reduced *H. parasuis* killing at 7 days post-infection (46), while Oleksiewicz and Nielsen found that *E. coli* uptake was not reduced at 24 hours (20), indicating that bacterial species and infection time-point both shape which defect is measurable; whether early capture and late killing are distinct failure modes has not been resolved. Mechanistic studies in immortalized lines (3D4/21, MARC-145, HEK-293T) report effects that have only partially been confirmed in primary PAMs (1, 57), and at least one major axis, the proposed HOXD8 transcriptional regulation of ITGα5 (57), has not yet been independently replicated in PRRSV systems. These observations identify the variables (strain, species, time, system) that future work would need to control. These boundary conditions define when PRRSV–PAM should be interpreted as a bacterial-refuge model and when it is better understood as extracellular clearance failure.

## 3 Candidate upstream amplifiers

These antibacterial defects arise in a macrophage whose innate signaling, degradative trafficking, metabolism, and lipid homeostasis are remodeled by PRRSV. Whether these remodeled host programs amplify antibacterial failure depends on how they alter bacterial fate.

### 3.1 Viral signaling inputs to macrophage attrition and cytokine misdirection

PRRSV does not uniformly silence macrophage signaling. It changes the timing and cellular distribution of pathways that coordinate antiviral defense, inflammatory injury, and recruitment of other antibacterial effector cells. The antibacterial interpretation is most direct for cytokine relay, but most studies still measure host signaling rather than bacterial clearance in the same experiment.

nsp1β interacts with G3BP1 (via residues VS19GG) to redirect the antiviral stress-granule response into a pro-viral platform that antagonizes PKR activation and its downstream NF-κB-dependent cytokine production, thereby suppressing TNF-α, IL-6, and CXCL8 synthesis in primary PAMs (58). Separately, multiple viral proteins activate MALT1-dependent NF-κB signaling early in infection, whereas nsp6 suppresses the same pathway at later time points (59). CXCL8 is a major neutrophil chemoattractant, while TNF-α supports inflammatory recruitment and activation. Whole-blood time-series deconvolution shows that PRRSV alters monocyte- and neutrophil-associated inflammatory programs over time (60), but it does not establish a chemokine-to-recruitment causal chain. Direct mechanistic precedent comes from influenza recovery, where IFN-mediated suppression of neutrophil chemoattractants impaired *Streptococcus pneumoniae* (*S. pneumoniae*) clearance and exogenous chemokine restoration rescued defense (61).

PRRSV also disrupts two major antiviral sensing pathways: the RIG-I-like receptor (RLR)–IRF3 axis (62–65) and the cGAS–STING axis (50, 66) (**Figure 2**). These pathways primarily govern type I interferon production, so their antibacterial relevance is less direct than that of the NF-κB and PKR modules (67–69). The inflammatory phenotype is not simple silencing. In contrast to STING antagonism, PRRSV nsp5 and nsp2 activate NLRP3 pathways and promote IL-1β release or pyroptosis (50–52). These studies measured host signaling or death rather than bacterial clearance.

**Figure 2:**
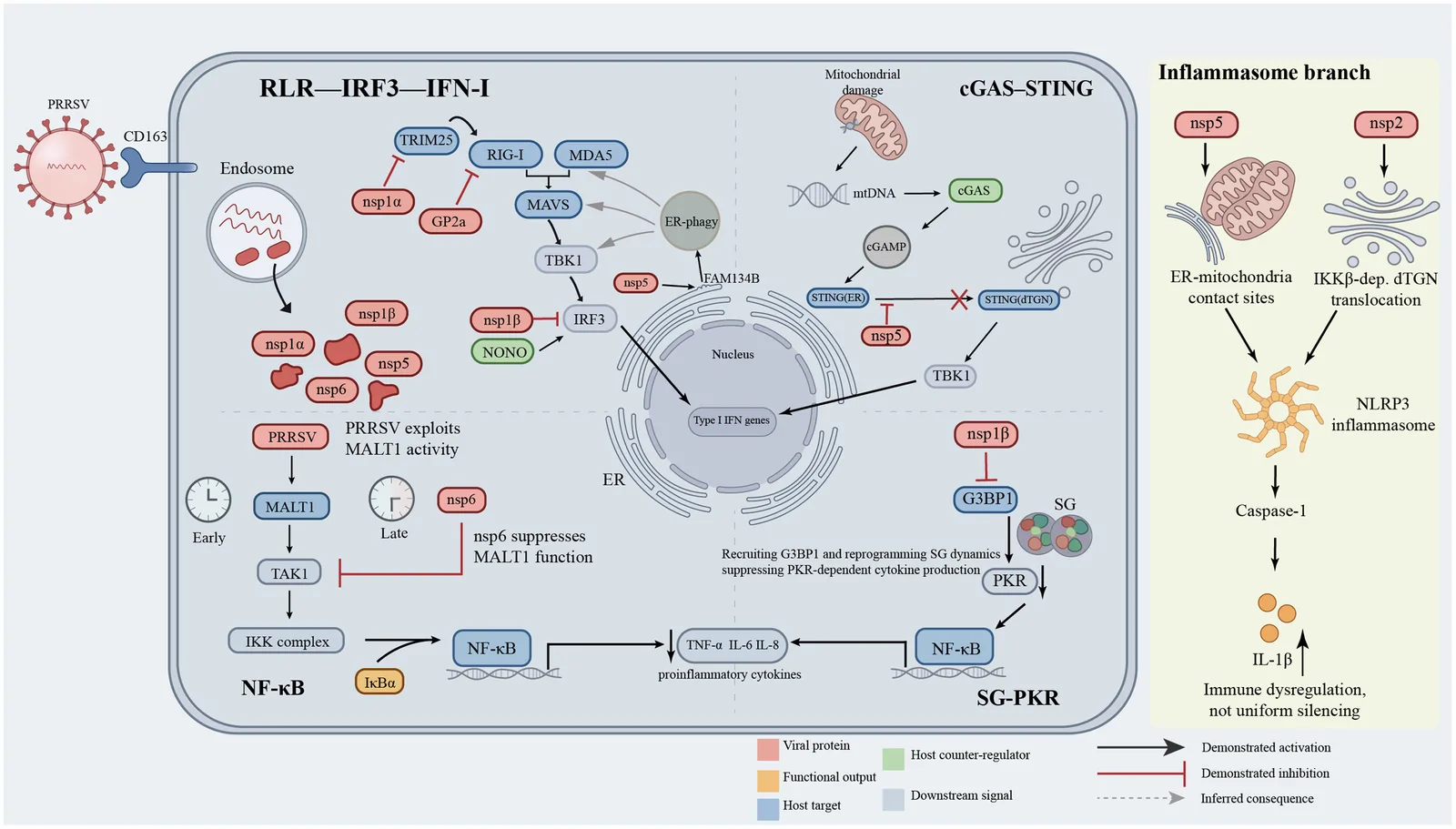
PRRSV-induced cytokine-relay disruption as a candidate amplifier of antibacterial failure. PRRSV non-structural proteins (red) selectively engage and re-wire host innate-immune modules (blue host targets; green host counter-regulators; grey downstream signals) rather than uniformly silencing them. (Top left, RLR–IRF3–IFN-I axis) After CD163-mediated entry and endosomal escape, nsp1α targets TRIM25 to suppress RIG-I ubiquitination, GP2a targets RIG-I, nsp1β inhibits IRF3 phosphorylation and nuclear translocation (with NONO as host counter-regulator), and nsp5 routes pathway components into FAM134B-mediated ER-phagy, collectively dampening type I IFN transcription. (Top right, cGAS–STING axis) Mitochondrial damage releases mtDNA that activates cGAS; cGAMP triggers STING translocation from ER to dispersed trans-Golgi network (dTGN); nsp5 retains STING at the ER, preventing TBK1 recruitment and type I IFN induction. (Bottom left, NF-κB axis with biphasic regulation) Early in infection PRRSV exploits MALT1→TAK1→IKK→NF-κB activation to drive proinflammatory cytokine transcription; later, nsp6 suppresses MALT1, reducing this output. (Bottom right, SG–PKR axis) nsp1β co-opts G3BP1 to reprogram stress-granule dynamics and suppress PKR-dependent NF-κB-driven production of TNF-α, IL-6, and CXCL8/IL-8. (Right inset, inflammasome branch) nsp5 acts at ER–mitochondria contact sites and nsp2 promotes IKKβ-dependent dTGN translocation; both converge on NLRP3 inflammasome activation, caspase-1 cleavage, and IL-1β release. Solid arrows indicate demonstrated activation; red bars indicate demonstrated inhibition; dashed grey arrows indicate inferred consequence.

Macrophage-state labels require similar restraint. PRRSV-associated M2-like shifts have been reported (70, 71), but their timing, marker profile, and relation to viral virulence vary. The antibacterial effect of Arg1 is supported by tuberculosis and toxoplasmosis models (72), not by a direct comparison of bacterial killing in PRRSV-associated PAM states. In one PRRSV-2 BALF single-cell dataset comparing the JA142 and NA10 strains, viral tracking classified fewer than 5% of macrophages as directly infected at the sampled time points, with the highest counts at JA142–7 dpi and NA10–3 dpi (53); this proportion is specific to the strains, sampling times, and classification used. The same study identified exosomal microRNAs with predicted targets in cell-death pathways, supporting a proposed bystander mechanism rather than demonstrating exosome-mediated killing. PD-L1, IDO1, and IL-10 were preferentially upregulated in productively infected PAMs relative to bystander cells (54). Together, these data support a heterogeneous population model in which infected and bystander macrophages occupy distinct regulatory states; Section 2.3 addresses how this heterogeneity may scale to antibacterial failure.

### 3.2 Autophagy–lysosome and xenophagy backup pathways

After the primary phagolysosomal killing pathway discussed above, autophagy-related pathways are best viewed as backup or auxiliary degradation systems whose antibacterial relevance depends on bacterial lifestyle and intracellular route. In xenophagy, bacteria that breach the phagosomal membrane and enter the cytosol are tagged with ubiquitin and delivered to autophagosomes for lysosomal destruction. Phagosome maturation and xenophagy use distinct fusion machinery but converge on lysosome-mediated degradation, making the latter a defined candidate backup pathway during PRRSV infection.

PRRSV targets the autophagic branch of this degradation. nsp5 disrupts the interaction between STX17 and SNAP29, two SNARE proteins required for autophagosome–lysosome docking, causing accumulation of immature autophagosomes in MARC-145 cells (73).

A separate branch involves chaperone-mediated autophagy (CMA), a pathway that translocates individual unfolded proteins (but not intact bacteria) across the lysosomal membrane (74). The viral structural protein GP5 inhibits CMA by targeting the lysosomal receptor LAMP2A (75), and RAB18 redirects CMA-associated lipolysis (76). CMA disruption is therefore relevant to bacterial defense only indirectly, through effects on lysosomal function and metabolic substrate availability and not through direct bacterial degradation. Additional observations, including host counter-regulation of p62 (77) and ER stress-driven pro-viral autophagy (78), further alter autophagic flux but without clear implications for bacterial handling.

If confirmed in PAMs, an autophagosome–lysosome fusion block would remove a xenophagy backup pathway alongside the RhoA-dependent phagolysosomal defect. This defines a specific paired test in PRRSV-infected primary PAMs: autophagic-fusion flux, bacterial localization, and bacterial survival measured in the same system.

### 3.3 Metabolic and lipid remodeling as candidate amplifiers

Metabolic and lipid remodeling form the most exploratory amplifier layer. PRRSV redirects macrophage metabolism through virus-directed glycolysis and calcium-mediated mitochondrial reorganization (79) and also reshapes lipid metabolism. The key question is which of these changes alters bactericidal metabolism or bacterial fate.

In PRRSV-infected macrophages, nsp1β stabilizes HIF-1α to promote viral replication (80), and the resulting glycolytic shift may prime lactylation of HSPA6 (81) to suppress IFN-β signaling. Sun et al. directly measured increased glycolytic activity and lactate production in PRRSV-infected primary PAMs, together with reduced oxidative phosphorylation (79). Cyclic pentose-phosphate fueling of NOX2 is established in activated neutrophils rather than macrophages (82), while gp91phox-null mice demonstrate the antibacterial importance of NOX2 (83). These observations support a concrete PAM experiment that measures carbon flux, NADPH/NADP^+^, NOX2 activity, and bacterial killing together.

PRRSV also reprograms lipid metabolism through nsp4-mediated ATGL degradation (84), SREBP-linked lipogenesis (85), and miRNA-directed remodeling of specific lipid species (86). Cross-system studies place this remodeling between opposing antibacterial possibilities: lipid-laden foamy macrophages can sustain mycobacterial persistence (87–89), whereas lipid droplets in activated macrophages can recruit antimicrobial proteins (90, 91). ROS elevation in primary PAMs also varies across studies (92). Lipid composition and activation context, not droplet abundance alone, are therefore the informative variables; paired lipidomic and bacterial-survival measurements in infected PAMs can distinguish permissive from protective remodeling.

## 4 Cytoskeletal and bacterial-side interfaces

PRRSV-associated defects in bacterial capture, killing, and inflammatory relay may converge with upstream remodeling at both cell and tissue levels. Bacterial access routes and Rho-family control nodes provide a basis for asking whether selective entry can coincide with impaired killing.

The population-level and cell-intrinsic frameworks are not mutually exclusive: population-level immune failure may create a permissive environment, while cell-intrinsic reprogramming may amplify bacterial survival within the infected minority. **Figure 1** summarizes the population-level and cell-intrinsic scales, whereas **Figure 3** displays the two candidate cell-intrinsic arms separately and the primary-PAM experiment needed to test their proposed combination.

**Figure 3:**
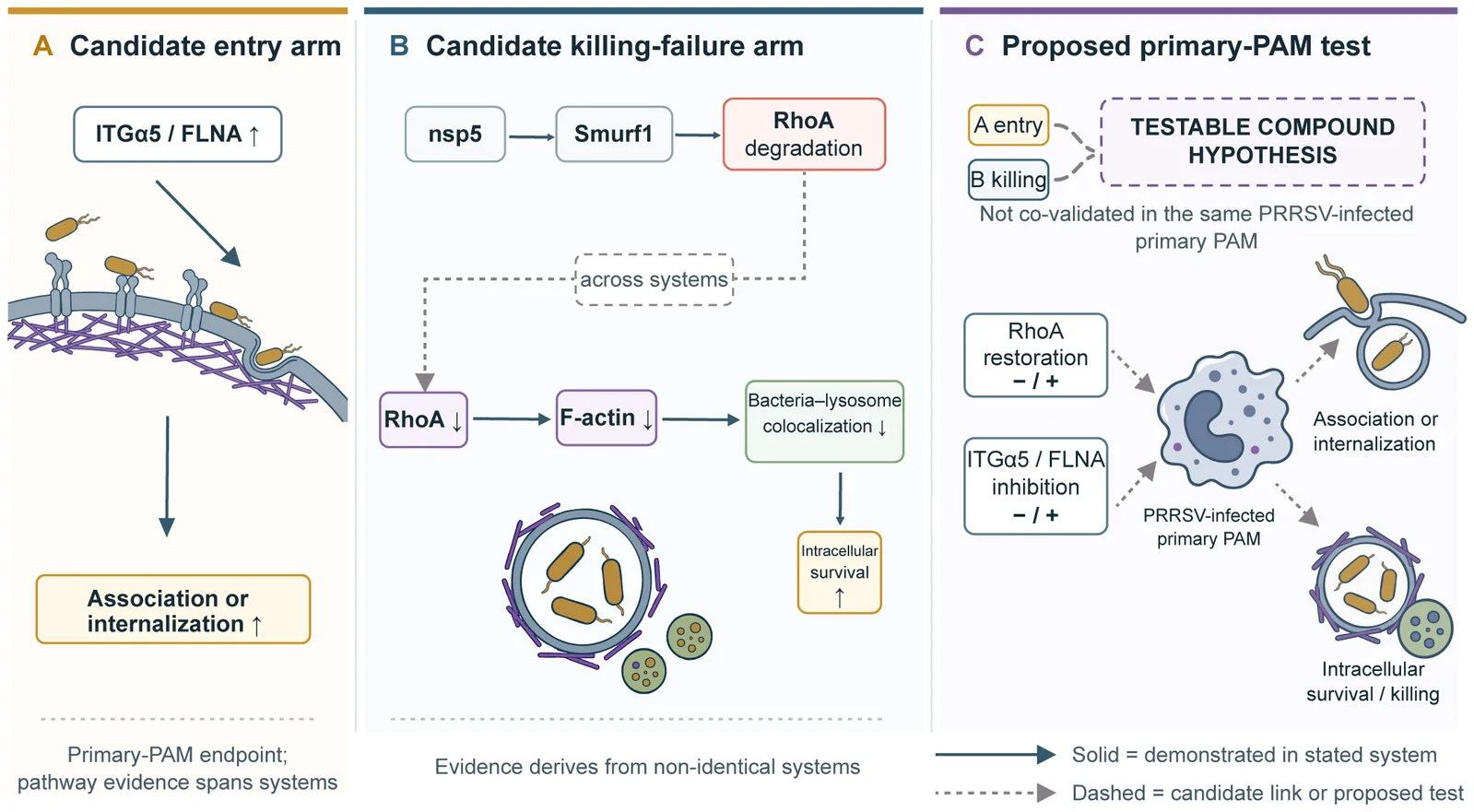
Candidate entry and killing-failure arms and a proposed primary-PAM test. **(A)** Candidate entry arm. PRRSV increases association or internalization of selected bacteria—Streptococcus suis, Klebsiella pneumoniae, and Aerococcus viridans—but not all tested species (57). A primary-PAM entry phenotype was observed, whereas much of the candidate ITGα5/FLNA pathway, including nsp10–HOXD8, was resolved in 3D4/21, MARC-145, or heterologous systems. Panel A combines components demonstrated in different systems; it does not depict a complete pathway validated in primary PAMs. **(B)** Candidate killing-failure arm. nsp5–Smurf1 promotes K63-linked ubiquitination and TOLLIP-dependent RhoA degradation. In Aerococcus viridans-challenged immortalized PAMs, RhoA knockdown reduced F-actin and bacteria–lysosome colocalization and increased viable bacteria, whereas overexpression produced reciprocal effects (1). These assays were separate from a PRRSV-context rescue, which remains untested in primary PAMs. **(C)** Proposed primary-PAM test. The two candidate arms have not been shown to coexist in the same PRRSV-infected primary PAM. A 2 × 2 design combining RhoA restoration with ITGα5/FLNA inhibition, while measuring bacterial entry and intracellular fate as separate, assay-specific endpoints, can distinguish interaction, independence, or failure of either arm. Entry is predicted to benefit bacteria only when downstream killing is impaired. The refuge model is most relevant to bacteria capable of conditional intracellular persistence; predominantly extracellular pathogens may instead benefit from population-level clearance failure (**Figure 1**). Slash-separated terms in panels A and C are assay-specific alternatives, not interchangeable endpoints: association differs from internalization, and survival from directly measured killing. Solid arrows denote relationships demonstrated in the stated experimental system. Dashed elements denote candidate cross-system links, untested integration, or proposed tests. The compound model is a testable hypothesis, not an established integrated mechanism.

### 4.1 Bacterial adhesion and respiratory-mucosal access as boundary-setting variables

Macrophage antibacterial failure becomes relevant only after bacteria reach the phagocyte compartment, but their routes to that compartment are pathogen-dependent. Some organisms first colonize mucus, epithelial surfaces, or exposed extracellular-matrix proteins and may then cross an epithelial barrier; inhaled bacteria can also encounter alveolar phagocytes more directly. Mucosal adhesion, barrier crossing, and direct alveolar contact should therefore be treated as parallel access routes rather than a universal sequence. Mucosal access, macrophage association, internalization, and intracellular survival are likewise biologically distinct, pathogen-dependent events.

*Streptococcus suis* illustrates how bacterial-side factors can connect these interfaces. Its anchorless fibronectin-binding protein FBPS has separable surface-association and host-cell/fibronectin-binding functions, although deletion of fbps does not abolish adhesion (93). This residual binding is consistent with partially redundant adhesins. A recent study of an *S. suis* serotype 2 strain identified Atl as a two-sided adhesion bridge: its Bsp-like domain binds bacterial lipoteichoic acid, whereas its C-terminal hydrolase domain binds epithelial fibronectin (94). Fibronectin-binding proteins can use fibronectin as a bridge to α5β1 integrin (93, 95, 96), making a bacterial adhesin–fibronectin–ITGα5 interface plausible. Whether this bridge operates in PRRSV-infected airway epithelium or PAMs remains unresolved, as does the breadth of Atl-mediated adhesion across serotypes.

*Glaesserella parasuis* defines a related barrier-level route. Serotype 4 HPS4-YC adhered to and invaded swine tracheal epithelial cells, reduced transepithelial resistance, lowered ZO-1 and occludin abundance, and increased bacterial passage; selected barrier changes were also observed in piglet lungs (97). A serotype 5 strain disrupted membrane replenishment of Claudin-1 through an autophagy-dependent process (98). Together, these studies show strain-specific epithelial adhesion, barrier disruption, and translocation in porcine respiratory systems. How these strain-specific epithelial effects shape PRRSV coinfection, PAM uptake, or intracellular killing remains unknown.

The ITGα5/FLNA study identifies a host-side macrophage interface that may operate after mucosal access or during direct alveolar contact. PRRSV increased adhesion or invasion of selected bacteria in cell and animal models, and ITGα5 or FLNA depletion reduced those endpoints (57). The bacterial ligands involved and any PRRSV-dependent changes in airway-epithelial receptors remain unknown. Macrophage association, internalization, and survival represent distinct stages of this process. Within a proposed bacterial-refuge model, increased macrophage entry becomes pro-bacterial only if the entering organism avoids or outlasts downstream killing; co-occurrence of the ITGα5/FLNA and RhoA arms has not been demonstrated in the same primary PAM. For predominantly extracellular pathogens, mucosal access or direct alveolar exposure followed by delayed capture and cytokine-relay failure may be more relevant than a stable intracellular niche.

### 4.2 Rho-family GTPases as context-dependent control nodes in bacterial infection

Several cell biological nodes recur across host–pathogen interactions involving phagocytes and help interpret the PRRSV findings integrated here. Rho-family small GTPases (RhoA, Rac1, Cdc42) regulate the actin assembly that drives phagocytic cup formation and pseudopod extension, and they continue to organize the cortical actin scaffold that supports phagosome maturation toward lysosomal fusion (38–40, 43). Rac1 and Cdc42 are especially prominent in phagocytic-cup protrusion and closure, whereas RhoA supports contractile actin organization and later phagosomal trafficking; the contribution of each member varies with the receptor and bacterial strategy (38–43). Integrin-mediated bacterial recognition is organized by filamin-cross-linked cortical actin, and many bacterial pathogens express fibronectin-binding proteins that engage this axis to gain entry (93, 95, 96). Phagosome maturation—the sequential fusion of phagosomes with progressively acidified compartments and ultimately lysosomes—is a major mechanism of intracellular bacterial killing (39, 43). Bacterial pathogens have repeatedly converged on these same nodes through evolutionarily distinct effectors, an observation described as convergence of pathogenicity evolution towards Rho GTPases (41, 42). The PRRSV-induced lesions summarized in Sections 2.1–2.4—reduced phagocytic capture, impaired phagosomal killing through nsp5-driven RhoA destruction, and integrin–FLNA-mediated entry enhancement—operate at these same nodes. The receptor, host cell, bacterial strategy, and measured endpoint determine whether Rho-family activity favors capture, entry, or post-entry trafficking.

RhoA-dependent actin remodeling contributes broadly to phagosomal trafficking, so this lesion could impair lysosomal delivery for a range of bacteria entering through conventional phagocytosis. This prediction is consistent with the multiple species affected in the original study (1) but has not been tested with a broader pathogen panel. That a viral effector targets the same GTPase node inactivated by *Yersinia* YopE/YopT (99) identifies RhoA as a recurring target in phagocyte manipulation across kingdoms. Bacterial pathogens covalently modify Rho-family GTPases through diverse mechanisms including ADP-ribosylation, glycosylation, and proteolysis (41). *Mycobacterium tuberculosis* achieves an analogous arrest of phagosome maturation through PI3P-pathway interference and a lysosomal-delivery block (100). Recent work further localizes this cytoskeletal connection: SIRT7 deficiency impairs RAC1-dependent actin remodeling in Mtb-infected macrophages, paralleling the PRRSV nsp5–RhoA–F-actin axis at the host node level (101). Tirap controls Mtb-phagosome acidification (102). The PRRSV nsp5–RhoA axis represents viral perturbation of a host node also targeted by bacteria across phyla, placing the finding within a broader framework of pathogen-induced disruption of phagocyte function.

Earlier work in cultured MARC-145 cells showed that intact actin and microtubule networks were required for PRRSV infection and cell-to-cell spread: colchicine and cytochalasin D reduced both primary infection and secondary cluster formation (103). These experiments concern viral dissemination in a permissive monkey kidney cell line, a setting distinct from later studies that linked defined cytoskeletal perturbations to antibacterial endpoints (1, 57).

Rho-family biology provides the principal link between PRRSV-induced cytoskeletal disruption and antibacterial failure, with nsp5 acting as the relevant viral perturbation. Among the reported nsp5 activities involving STING, RLR, NLRP3, autophagic fusion, Nrf2, and RhoA, the RhoA arm is the one paired with bacterial trafficking and viable-bacteria endpoints in the same study. The antibacterial consequences of these other nsp5 activities remain open questions (**Table 1**).

**Table 1:** Documented nsp5–host pathway activities and their antibacterial relevance.

Activity	Host target/experimental system	Measured host outcome	Bacterial endpoint in the same experiment	Validation/limitation	Ref
cGAS–STING antagonism	STING; PRRSV-permissive and overexpression systems	ER retention and reduced IFN-I signaling	None	Residues 36–47 and 58–67 mapped; antibacterial consequence untested	(50, 66)
RLR suppression	RIG-I–IFN axis; cell-line and overexpression systems	Dampened antiviral signaling	None	Domain unmapped; no paired bacterial test	(62)
NLRP3 activation	NLRP3; PAM and infectious-clone evidence	IL-1β release and pyroptosis	None	G30A/N35A mutants inform host-cell phenotype; bacterial consequence untested	(50)
Autophagic-fusion block	STX17–SNAP29; MARC-145	Immature autophagosome accumulation	None	Primary PAMs and bacterial coinfection untested	(73)
Nrf2 suppression	p62–Keap1; PRRSV cell systems	Impaired antioxidant program	None	Y146/R147 mutant evidence; no paired bacterial endpoint	(77)
RhoA degradation	RhoA via Smurf1; mainly 3D4/21-CD163 with partial PAM validation	F-actin loss and impaired lysosomal delivery	Viable intracellular bacterial recovery and bacteria–lysosome colocalization	Candidate mechanism with complementary RhoA gain- and loss-of-function data; PRRSV-context rescue and independent primary-PAM replication remain pending.	(1)

**Table 1** summarizes documented nsp5–host pathway activities, the experimental systems in which they were studied, and whether a bacterial endpoint was measured in the same experiment.

Only the RhoA row includes a bacterial CFU and lysosomal-delivery endpoint in the same study; the other rows summarize host-pathway perturbations whose antibacterial effects remain untested.

### 4.3 Entry-plus-killing compound failure as a testable model

Available phenotypic studies support two cell-level outcomes: reduced bacterial killing or increased intracellular survival (1, 46, 47), and increased association or internalization of selected bacteria (47, 57). Separate animal coinfection studies report higher bacterial burdens or more severe disease under defined conditions (13–16, 47), without assigning those outcomes to either cellular arm. Together, these observations motivate a compound-failure hypothesis: increased association or entry could raise bacterial exposure to an intracellular environment whose killing capacity is reduced. The molecular candidates for each arm—nsp5-mediated RhoA degradation (1) and ITGα5/FLNA upregulation (57)—were identified in separate studies, so the model is defined by the two phenotypes and can be tested without presupposing either exact molecular route.

The model has a directional constraint: enhanced entry into a killing-competent macrophage would accelerate clearance, whereas bacterial benefit requires a concurrent downstream killing defect. Testing both endpoints in a shared primary-PAM system would determine whether FLNA-dependent cortical scaffolding and RhoA-dependent trafficking coexist and interact.

In this framework, a bacterial refuge arises when entry into a reprogrammed macrophage benefits the bacterium, whereas clearance failure results from impaired capture, killing, or inflammatory relay. *K. pneumoniae* is included as an experimental organism in mechanistic studies (1, 57), not as a primary clinical partner in PRRSV coinfection. The refuge model is most plausible for bacteria capable of conditional intracellular persistence, whereas predominantly extracellular pathogens may benefit mainly from delayed population-level clearance.

## 5 Experimental priorities and future translational possibilities

### 5.1 Experimental priorities

The experimental priority is to pair viral lesions, macrophage functional state, and bacterial endpoints in the same primary PAM system.

The reproducible antibacterial phenotype now provides a foundation for resolving the underlying mechanisms. The nsp5–RhoA and ITGα5/FLNA studies provide specific starting points; testing them alongside endpoint-resolved phenotypes in independent primary-PAM systems will connect mechanism to the broader coinfection literature.

A systematic functional characterization should measure phagocytic uptake, intracellular killing, and cytokine output in primary PAMs after PRRSV infection, ideally with a panel of clinically relevant bacterial species. Pairing viral infection with host lesion and bacterial endpoint within the same experimental system, measuring bacterial entry and intracellular survival separately, including rescue or perturbation of the candidate host lesion, and comparing across PRRSV strains and genotypes would tighten the inference chain. Together, these measurements would establish a quantitative baseline for testing individual viral effectors. Coupling these readouts to single-cell transcriptomics (53) would stratify PRRSV-infected PAMs by viral load and resolve heterogeneity across the cell population.

One informative test would be a 2 × 2 factorial rescue in primary PAMs: restore RhoA signaling on one axis, block ITGα5–FLNA on the other, and measure both bacterial entry and intracellular survival. Single-arm rescue would test partial dependency; the factorial design can distinguish interaction from independence. If RhoA restoration leaves intracellular survival unchanged after entry is controlled, the data would favor a killing pathway outside this axis; if ITGα5–FLNA blockade changes adherence without altering intracellular fate, it would support component independence rather than synergy. Uniform pathogen responses across entry routes would likewise favor a general clearance-failure model over the refuge/clearance distinction.

Candidate upstream amplifiers require similarly paired tests. In primary PAMs, STX17–SNAP29 flux should be linked to bacterial localization and survival; carbon flux and NADPH/NADP^+^ availability should be examined alongside NOX2 activity and bacterial killing; and exosomal microRNA-mediated bystander death should be tested by donor-to-recipient transfer, blockade, and rescue. Infectious-clone nsp5 mutants can separate STING antagonism, NLRP3 activation, autophagic fusion, Nrf2 regulation, and Smurf1 recruitment, but each mutant must be evaluated for viral fitness before a bacterial phenotype is attributed to one host pathway.

PRRSV-1 versus PRRSV-2 comparisons in primary PAMs with parallel measurement of capture, killing, entry, and upstream markers (2, 53) would define strain dependence. The same design can compare virulence variants and test whether modified live vaccine strains perturb macrophage antibacterial defense to a lesser extent (56, 104).

Finally, which bacterial species gain a measurable survival advantage inside the reprogrammed macrophage, and which benefit primarily from delayed extracellular clearance? Testing these predictions with a broader pathogen panel would determine whether the bacterial refuge and clearance failure distinction holds.

### 5.2 Future translational possibilities

These possibilities remain experimental and require validation in field coinfection settings.

CD163 editing represents the clearest upstream proof of principle: SRCR5-domain deletion abolishes PRRSV entry while preserving hemoglobin–haptoglobin scavenging function, complementing earlier full-knockout strategies that confer heritable resistance without major performance penalties. Whether SRCR5 deletion preserves antibacterial CD163 functions, including bacterial recognition, has not been comprehensively assessed.

CD163 editing addresses prevention; a complementary question is whether macrophage antibacterial defense can be preserved or restored after infection has begun. In bacterially challenged immortalized PAMs, RhoA gain-of-function increased F-actin and bacteria–lysosome colocalization and reduced viable intracellular bacteria (1), establishing the experimental tractability of this host node. The corresponding post-infection test is to restore RhoA during PRRSV infection and measure bacterial entry and intracellular survival separately in primary PAMs. The nsp1β/N double mutant reduced inflammatory output and *Streptococcus suis* coinfection severity, but its lower viral fitness prevents attribution to a single protective mechanism (56).

Mapped nsp5 regions required for STING interaction (residues 36–47 and 58–67) provide experimental handles for testing recovery of STING signaling (50, 66). Mapping and selectively disrupting the nsp5–Smurf1 interface would provide a parallel test of RhoA-dependent trafficking (1). Because Smurf1 supports *Mycobacterium tuberculosis* xenophagy in macrophages (105), pathway-selective disruption of the viral interaction is preferable to broad Smurf1 inhibition. ITGα5/FLNA modulation can be evaluated at the same proof-of-concept stage, with bacterial entry and intracellular survival measured separately (57). These axes define experimental priorities for primary PAMs and natural-host coinfection models.

## 6 Discussion

PRRSV-associated antibacterial dysfunction is a convergent phenotypic observation documented across multiple independent laboratories and spanning three decades (19, 21, 45, 46). The nsp5–RhoA and ITGα5/FLNA pathways (1, 57) offer candidate molecular links to two established cell-biological nodes: Rho-GTPase control of phagosome maturation and integrin–filamin organization of bacterial uptake (38–40, 43). Innate immune dysregulation and autophagy–lysosome remodeling provide plausible upstream amplifiers, whereas the antibacterial effects of ER–mitochondrial metabolic shifts and lipid reprogramming remain unresolved. Together, these observations situate PRRSV–PAM among the growing set of virus–macrophage systems in which viral infection or exposure compromises antibacterial immunity (29, 36, 37).

The PRRSV–PAM relationship is one example of a broader host–pathogen pattern: viruses or viral exposures can reshape the cellular machinery underlying antibacterial defense in professional phagocytes. Analogous conflicts operate in influenza (22–25, 111), HIV–tuberculosis (26, 106–108) and SARS-CoV-2 (27, 28). HRV16 compromises macrophage bacterial clearance through both a cytokine-response defect (109), recently mechanistically traced to an ICAM-1-PKR-ATF2 axis (112), and a phagolysosome-biogenesis defect mediated by virus-induced ARL5b upregulation (110). ASFV reshapes the autophagy–lysosome axis in PAMs (113) and enhances macrophage phagocytosis of *E. coli* and *G. parasuis* through cGAS/STING–NF-κB-driven CD14 upregulation (114), contrasting with the reduced bulk capture reported in selected PRRSV systems (6, 19, 45). Together, these comparisons illustrate that myelotropic viruses can either impair or enhance macrophage antibacterial defense according to the host machinery they co-opt, rather than through a uniform “immune evasion” program. **Table 2** summarizes these virus–phagocyte contexts and their measured antibacterial outcomes.

**Table 2:** Endpoint- and system-specific comparison of virus-associated bacterial vulnerability in professional phagocytes.

Virus/context	Experimental system	Measured antibacterial endpoint	Host change or candidate mechanism	Claim status for stated endpoint	Key limitation
PRRSV: capture/macrophage availability	Primary PAMs, porcine monocyte-derived macrophages, and pig pulmonary-clearance models	Reduced bead, zymosan, or particle uptake/clearance in selected systems	Immediate receptor-linked capture impairment and later loss of functional phagocytes	Observed for stated particle or clearance endpoint	Particle uptake is not live-bacterial killing; effects vary by strain, time, cell source, and viable-cell denominator
PRRSV: intracellular killing arm	*Ex vivo* PAM killing studies; mainly 3D4/21-CD163 for PRRSV/nsp5 perturbation and separate RhoA loss- and gain-of-function assays	Reduced bactericidal activity, increased viable intracellular bacteria, impaired bacteria–lysosome colocalization	nsp5–Smurf1–RhoA–F-actin	Killing phenotype observed; molecular chain is a candidate mechanism	PRRSV-to-RhoA/F-actin and RhoA-to-bacterial-trafficking evidence were generated mainly in an immortalized model; a PRRSV-context rescue in primary PAMs remains the defining next test.
PRRSV: association/internalization arm	Primary PAM and animal endpoints; 3D4/21, MARC-145, and HEK-293T for much of the molecular chain	Increased association or internalization of selected bacteria	nsp10–HOXD8–ITGα5/FLNA	Selected entry endpoints observed; complete chain remains candidate	Species-dependent; systems and endpoints differ; no same-cell integration with RhoA arm
PRRSV: population route	Pig BALF or lung studies and defined coinfection models	Bacterial burden, pathology, or disease severity in defined coinfections	Macrophage attrition, state shifts, and cytokine misdirection	Animal outcomes observed; mechanistic contribution unresolved	Viral positivity, cell loss, cytokines, and bacterial outcomes are rarely paired; exosomal-miRNA route remains proposed
HIV–macrophage	Human monocyte-derived macrophages	Impaired phagocytosis of *M. avium*, *T. gondii*, and opsonized particles	Nef–AP1 recycling-endosome delivery; Tat–PI (4, 5)P_2_–Cdc42 cup recruitment	Direct for named phagocytic endpoints	Distinct pathogens and models; not evidence for the PRRSV molecular chain
HRV16–macrophage	Human monocyte-derived macrophages with non-productive viral exposure	Defective bacterial clearance and phagolysosome biogenesis	ARL5b induction and altered cytokine response	Direct for stated macrophage endpoints	Productive macrophage replication is not required; anatomical and viral context differs from PRRSV
Influenza–alveolar macrophage	Mouse and respiratory infection models; restrictive macrophage replication	Post-influenza bacterial pneumonia and impaired clearance	IFN-γ–MARCO, NOX2 suppression, and macrophage depletion	Direct or causal within the cited influenza models	Mechanism is not primarily a PRRSV-like cytoskeletal lesion
SARS-CoV-2–alveolar macrophage	Human and experimental lung studies	Secondary bacterial pneumonia association; altered lysosomal and macrophage-population states	Reduced lysosomal acidification and resident-to-monocyte-derived macrophage shift	Mixed: host changes observed; bacterial link partly indirect	Limited paired cellular bacterial endpoints
ASFV–PAM	Productively infected PAMs	Enhanced *Escherichia coli* and *Glaesserella parasuis* uptake	cGAS/STING–NF-κB-driven CD14 upregulation	Direct for stated uptake endpoint	Single primary study; opposite direction to PRRSV capture phenotype

“Direct” applies only to the endpoint named in a row; it does not imply independent replication or complete validation in primary cells. Candidate mechanisms and hypotheses are labeled separately.

By anchoring broader cross-virus analyses of macrophage programming (36) on antibacterial-defense endpoints, this review distinguishes shared principles from PRRSV-specific mechanisms within a tractable natural-host coinfection system. The broader implication is that productive macrophage infection can reshape antibacterial competence during virus–bacterium coinfection.

The phenotypic case for PRRSV-associated bacterial vulnerability is strong, while mechanistic resolution now depends on distinguishing causal lesions from context-dependent associations and determining which findings are conserved across strains and pathogens. Looking beyond PRRSV, these answers could inform host-directed strategies for secondary bacterial pneumonia after respiratory viral infection in veterinary and human medicine, where antibiotic options are increasingly constrained by resistance and preserving macrophage antibacterial function could provide a complementary intervention point.

The PRRSV–PAM system aligns productive infection, professional phagocyte biology, and bacterial challenge in the natural host, providing a tractable setting to examine how viral infection reduces bacterial clearance at cellular and tissue levels. The node-specific framework identifies discrete molecular lesions within infected macrophages while placing them alongside macrophage depletion, bystander dysfunction, and cytokine-relay failure at the tissue level. By linking defined antibacterial phenotypes to cytoskeletal lesions and population-level processes, this framework positions the PRRSV–PAM axis as a practical natural-host model of virus-associated bacterial vulnerability.
