# Supplementary material for: Cytoskeletal and phagolysosomal failure in macrophage antibacterial immunity: the PRRSV–porcine alveolar macrophage axis as a natural-host model
Source: Front Immunol. 2026 Oct 14;17:1892313. doi: 10.3389/fimmu.2026.1892313 (PMC13714797; doi:10.3389/fimmu.2026.1892313)
Supplement: Supplementary file 1 [file Table1.docx]

# Supplementary Materials

*Reference numbers correspond to the reference list in the revised manuscript. “Direct” applies only to the endpoint measured in the cited experiment; candidate and inferred antibacterial implications are identified explicitly.*

**Supplementary Table 1.** PRRSV-linked host perturbations discussed in this Review. The table summarizes the viral effector, host target, measured host outcome, experimental system, and current reference. Only the RhoA row includes bacterial CFU and lysosomal-delivery endpoints in the same study. The ITGα5/FLNA row includes selected bacterial association or invasion endpoints, whereas most other rows report host-pathway outcomes without a paired bacterial test.

| **Mechanism** | **Viral or Host Factor** | **Host Target** | **Measured Host Outcome** | **Experimental System** | **Refs** |
| --- | --- | --- | --- | --- | --- |
| Impaired particle uptake or pulmonary clearance | — | Sialoadhesin-dependent virion interaction; other targets unresolved | Reduced bead/zymosan uptake or pulmonary particle clearance; live-bacterial killing not measured | Primary PAMs, porcine monocyte-derived macrophages, and pig pulmonary-clearance model | (6, 19, 45) |
| RhoA degradation and lysosomal-delivery failure | nsp5 | Smurf1→RhoA→F-actin | Reduced F-actin and bacteria–lysosome colocalization; increased viable intracellular bacteria | Mainly 3D4/21-CD163; selected molecular steps in primary PAMs and piglets | (1) |
| Selective bacterial association or internalization | nsp10 (candidate HOXD8 route) | ITGα5, FLNA | Increased association or internalization of selected bacteria | Primary PAMs, 3D4/21, MARC-145, piglets, and a bacterial-only mouse model | (57) |
| RLR–IRF3 suppression | nsp5, nsp1α, nsp1β, GP2a | Multiple RLR components, TRIM25, IRF3, RIG-I | Reduced RLR–IRF3–IFN-I signaling; no paired bacterial endpoint | Cell-line and overexpression systems | (62–65) |
| cGAS–STING antagonism | nsp5 | STING | ER retention of STING and reduced IFN-I signaling; no paired bacterial endpoint | PRRSV-permissive and overexpression systems | (50, 66) |
| Dynamic MALT1 manipulation | Multiple nsps (early); nsp6 (late) | MALT1 | Early NF-κB activation followed by later suppression; no paired bacterial endpoint | Primary PAMs and 3D4/21 | (59) |
| SG–PKR axis disruption | nsp1β | G3BP1, PKR | Suppressed PKR-dependent NF-κB output and TNF-α/IL-6/CXCL8 production; no paired bacterial endpoint | Primary PAMs | (58) |
| NLRP3 inflammasome activation | nsp5, nsp2 | NLRP3, IKKβ | IL-1β release and pyroptosis; bacterial clearance not measured | Primary PAMs, 3D4/21, and infectious-clone evidence | (50–52) |
| GSDMD-dependent pyroptosis | — | GSDMD | PAM death; contribution to population-level antibacterial failure remains inferred | Primary PAMs | (51) |
| Study-specific M2-like marker changes | N, nsp1α, and nsp10-associated programs | Study-specific marker panels; Arg1 | Marker changes vary by strain, timing, and macrophage population; antibacterial consequence inferred from non-PRRSV models | PAM studies; antibacterial Arg1 evidence from tuberculosis and toxoplasmosis models | (70–72) |
| Autophagosome–lysosome fusion block | nsp5 | STX17–SNAP29 | Immature autophagosome accumulation; bacterial consequence untested | MARC-145 and HEK-293T | (73) |
| CMA inhibition | GP5 | LAMP2A, GFAP | CMA suppression and enhanced IFN-I evasion; intact bacteria are not CMA cargo | HEK-293T, PAMs, MARC-145, and 3D4/21 | (75) |
| CMA-linked lipolysis redirection | RAB18 (host) | HSPA8, PLIN2 | CMA-associated lipolysis redirected to support viral replication; antibacterial consequence untested | MARC-145, PAMs | (76) |
| ER stress–calcium–autophagy | — | SOCE/CaMKII/AMPK | Pro-viral autophagy induction; bacterial consequence untested | MARC-145, PAMs | (78) |
| Mitochondrial fission/mitophagy | — | Calcium/DRP1 | Glycolytic shift and lactate production; bacterial fate untested | MARC-145 and primary PAMs | (79) |
| Mitochondrial dysfunction | — | Multiple mitochondrial and electron-transport targets | Mitochondrial dysfunction with study-dependent ROS/NO changes; no paired bacterial endpoint | PRRSV-2-infected porcine macrophages | (92) |
| Nrf2/HO-1 suppression | nsp5 | p62–Nrf2 | Impaired antioxidant program; no paired bacterial endpoint | PAMs, MARC-145 | (77) |
| Lipid-droplet accumulation | nsp4 | ATGL | ATGL degradation and lipolysis blockade; bacterial consequence untested | MARC-145, PAMs | (84) |
| Lipid biosynthesis activation | — | SREBPs/AKT/PCK1 | ROS-dependent lipogenesis supporting viral replication; bacterial consequence untested | MARC-145, PAMs | (85) |
| Host restriction of lipid reprogramming | let-7f-5p (host) | SREBP2, lipid droplets | Suppressed PRRSV-driven lipid-droplet accumulation; antibacterial consequence untested | PK15-CD163, MARC-145 | (86) |

**Supplementary Table 2.** Clinical PRRSV coinfection bacteria and an experimental organism mapped to candidate clearance-failure and bacterial-refuge models. Observed endpoints are distinguished from candidate mechanisms; the two cytoskeletal arms were not co-validated in one primary PAM system.

| **Pathogen** | **Ecology / Intracellular Behavior** | **Candidate Failure Mode** | **Candidate Mechanism / Boundary** | **PRRSV–Bacterium Evidence / Endpoint** | **Key Assay(s)** | **Refs** |
| --- | --- | --- | --- | --- | --- | --- |
| *G. parasuis* | Predominantly extracellular; strain-dependent epithelial invasion or translocation reported | Clearance failure supported; PRRSV-dependent refuge not established | Reduced PAM post-entry clearance; altered macrophage viability and cytokine relay | Piglets: increased pathology and load in one HP-PRRSV2 model; ex vivo PAMs: reduced clearance; macrophages: altered viability or cytokines. Refs 97–98 support epithelial behavior only. | Pathology or load; time-resolved PAM CFU; viability or cytokines | (15, 46, 49, 55, 97, 98) |
| *S. suis* | Predominantly extracellular; model-dependent intracellular survival reported | Clearance failure primary; refuge is a conditional hypothesis | Separate candidate ITGα5/FLNA entry and nsp5–RhoA killing-failure arms; cytokine dysregulation | Pig models show condition-dependent susceptibility, severity, or burden; Ref. 47 reports accelerated infection in vivo and in vitro. Cytoskeletal arms were not co-validated in primary PAMs. | Burden or pathology; gentamicin protection separating association, internalization, survival, and killing | (1, 13, 14, 16, 47, 56, 57) |
| *A. pleuropneumoniae* | Predominantly extracellular; uptake can occur; no established intracellular niche | No mechanism assigned | No pathogen-specific PRRSV capture, entry, or killing lesion assigned | Current reference set supports PRDC or clinical context only; Ref. 12 does not establish a macrophage mechanism. | Pathogen-specific controlled coinfection and macrophage assays needed | (12) |
| *K. pneumoniae* | Classically extracellular; conditional survival in macrophage models | Both as candidate models; not integrated | Separate candidate nsp5–RhoA killing-failure and ITGα5/FLNA entry arms; no primary-PAM integration | Experimental PRRSV model only: Ref. 1 measured viable intracellular K. pneumoniae; Ref. 57 tested entry separately. No natural-host clinical evidence. | Gentamicin protection separating entry, survival, and killing; CFU | (1, 57) |

*Note: “Predominant ecology” does not exclude transient uptake or short-lived intracellular survival. “Bacterial refuge” denotes a candidate benefit after entry into a reprogrammed macrophage; “clearance failure” includes impaired capture, killing, macrophage availability, or inflammatory relay. Intracellular refuge has not been established as PRRSV-dependent for S. suis, G. parasuis, or A. pleuropneumoniae. K. pneumoniae is experimental only. The RhoA and ITGα5/FLNA arms were reported separately and were not co-validated in PRRSV-infected primary PAMs.*

**Supplementary Table 3.** Candidate prevention and post-infection strategies relevant to macrophage antibacterial defense during PRRSV infection. All remain experimental for bacterial coinfection, and none has been validated in field settings; related caveats are discussed in Section 5.2.

| **Strategy** | **Target type** | **Specific target** | **Status** | **Risk / Caveat** | **Refs** |
| --- | --- | --- | --- | --- | --- |
| CD163 SRCR5-domain editing | Viral-entry prevention | CD163 SRCR5 domain | PRRSV resistance and heritability demonstrated in pigs | Effects on antibacterial CD163 functions have not been comprehensively assessed | (8, 10) |
| Full CD163 knockout | Viral-entry prevention | CD163 | PRRSV resistance demonstrated in pigs | Removes all CD163 functions; antibacterial consequences remain unresolved | (7) |
| RhoA pathway restoration | Host cytoskeletal pathway (post-infection) | RhoA–F-actin–lysosomal delivery | Gain-of-function improved bacterial-trafficking and viable-bacteria endpoints in immortalized PAMs | Not a PRRSV-context rescue; primary-PAM validation remains pending | (1) |
| Broad Smurf1 inhibition | Host E3 ligase | Smurf1 | Conceptual; no PRRSV-specific inhibitor validation | May impair Smurf1-dependent xenophagy | (1, 105) |
| Selective disruption of the nsp5–Smurf1 interaction | Viral-host interface | nsp5–Smurf1 interaction | Conceptual; no antagonist has been reported | Interface mapping, selectivity, and viral-fitness effects require testing | (1) |
| ITGα5/FLNA modulation | Host entry/scaffold pathway (post-infection) | ITGα5 and FLNA | Genetic perturbation altered bacterial adhesion or invasion in experimental systems | No PRRSV-directed pharmacologic intervention; homeostatic integrin functions may be affected | (57) |

*Abbreviations: CFU, colony-forming unit; CMA, chaperone-mediated autophagy; FLNA, filamin A; IFN-I, type I interferon; ITGα5, integrin α5; PAM, porcine alveolar macrophage; PIM, pulmonary intravascular macrophage; PRDC, porcine respiratory disease complex; RLR, RIG-I-like receptor; SG, stress granule.*
